# Correction to ‘Self-induced class stratification in competitive societies of agents: Nash stability in the presence of envy’

**DOI:** 10.1098/rsos.201626

**Published:** 2020-10-14

**Authors:** Claudius Gros

**Keywords:** game theory, social modelling, class separation, social stratification

*R. Soc. Open Sci.* 7, 200411. (Published 17 June 2020). (doi:10.1098/rsos.200411)

The argumentation regarding the derivation of the theoretical description of the class stratified state was not fully consistent. The respective amendment is presented. All analytical and numerical results remain untouched.

We correct the line of arguments used for the derivation of the theoretical description of the class stratified state. The notion of a lock-in condition was missing in particular. We recall, as defined in §4.1, that *q*_U_ and *q*_¬U_ denote options within the lower-class support played/not played by upper-class agents. The second paragraph in §4.1 should read as follows:

Central to our considerations are the properties of evolutionary stable strategies [38], in particular that the pay-off is constant within the support. For *q*_*i*_ = *q*_¬U_ for which *p*_L_(*q*_*i*_) > 0 and for which all upper class policies vanish, *p*_U_(*q*_*i*_) = 0, the upper class pay-off *E*^U^(*q*_¬U_) is

$$\begin{array}{rl} E^{U}(q_{\neg U}) & =v(q_{\neg U})-\kappa M_{L}p_{L}(q_{\neg U})\\ R_{L}-E^{U}(q_{\neg U}) & =p_{L}(q_{\neg U})[\kappa +\varepsilon \log(R_{L}/\bar{R})], \end{array}$$

where we used *R*_L_ = *v*(*q_¬_*_U_) − *κ*[*M*_L_ − 1]*p*_L_(*q*_¬U_) + *ε p*_L_(*q*_¬U_) $\log(R_{L}/\bar{R})$ to eliminate *v*(*q*_¬U_). The lower-class strategy is evolutionary stable, which implies that the left-hand side of the second equation is positive. The term $\log(R_{L}/\bar{R})$, which is negative for the lower class, drives the right-hand side however to negativity, with increasing envy, which is not allowed. The class stratification transition is hence given by the lock-in condition *R*_L_ = *E*^U^(*q*_¬U_).

The paper has now been updated.

